# Glucose metabolic reprogramming in systemic lupus erythematosus and lupus nephritis: theoretical foundations and therapeutic implications

**DOI:** 10.3389/fimmu.2026.1799232

**Published:** 2026-04-02

**Authors:** Hongyong Su, Le Zhang, Qiaofei Zhang, Lijing Liu, Liping Zhai, Xiaocui Chen, Chen Yang, Ning An, Hua-feng Liu

**Affiliations:** 1 Department of Nephrology, National Clinical Key Specialty Construction Program (2023), Institute of Nephrology, Guangdong Provincial Key Laboratory of Autophagy and Major Chronic Noncommunicable Diseases, Key Laboratory of Prevention and Management of Chronic Kidney Disease of Zhanjiang City, Affiliated Hospital of Guangdong Medical University, Zhanjiang, China; 2 Faculty of Chinese Medicine, State Key Laboratory of Quality Research in Chinese Medicine, Macau University of Science and Technology, Macao, Macao SAR, China; 3 Guangdong Medical University, Zhanjiang, China

**Keywords:** glucose, glycolysis, immune cells, metabolic reprogramming, systemic lupus erythematosus

## Abstract

Lupus nephritis (LN) represents the most severe and frequent complication of systemic lupus erythematosus (SLE), yet its treatment remains a significant unmet clinical need. Recent advances in immunometabolism have revealed that glucose metabolic reprogramming—including shifts in glycolysis, the pentose phosphate pathway (PPP), and the tricarboxylic acid (TCA) cycle—plays a central role in driving pathogenic immune cell activation in SLE. However, a critical gap persists in understanding how these metabolic alterations specifically operate within the renal microenvironment to promote immune cell infiltration and intrinsic kidney cell injury in LN. This review synthesizes current evidence on the molecular mechanisms linking glucose metabolism to immune dysfunction in innate immune cells including monocytes/macrophages, neutrophils and DCs and adaptive immune cells including T cells, B cells and renal resident cells. We further discuss therapeutic strategies targeting metabolic pathways, including repurposed drugs (metformin, hydroxychloroquine, rapamycin), preclinical small molecules (PKM2, PFKFB3, LDHA, GLUT1 inhibitors), and combination therapies with biologics. Safety considerations, particularly the sensitivity of regulatory T cells (Tregs) to glycolysis inhibition, underscore the need for dose optimization. Finally, we highlight future directions, including real-time metabolic imaging, personalized glycolysis scoring, and spatiotemporal metabolic epigenetic models, which hold promise for advancing precision medicine in LN.

## 1 Introduction

Lupus nephritis (LN) represents the most severe and frequent complication of systemic lupus erythematosus (SLE), affecting over 60% of patients during their disease course and constituting a major cause of morbidity and mortality. Approximately 7.9% of LN patients progress to end-stage renal disease, underscoring a critical unmet need for more effective and targeted therapies (1, 2). LN pathogenesis is rooted in the dysregulation of both innate and adaptive immunity within the kidney, driven by genetic, epigenetic, and environmental factors that promote systemic autoimmunity (3). While B cells secreting pathogenic autoantibodies are central to systemic damage, other immune cells—including T cells, macrophages, neutrophils, and dendritic cells (DCs)—infiltrate the kidney and perpetuate local inflammation (4). Our research group has also demonstrated that basophils contribute to SLE pathogenesis, though their direct role in LN requires further investigation (5). Beyond immune cell infiltration, renal resident cells such as podocytes, mesangial cells, and tubular epithelial cells actively participate in the injury response, creating a complex interplay between infiltrating leukocytes and the kidney microenvironment. Currently, LN treatment relies primarily on high-dose glucocorticoids and immunosuppressants, which are associated with significant adverse effects due to their potent and non-selective immunosuppressive properties (6). Glucocorticoids, in particular, cause profound endocrine and metabolic disturbances. Thus, there is an urgent need for novel immunomodulatory strategies that can control LN more precisely with fewer systemic toxicities.

In recent years, metabolic reprogramming has emerged as a central link connecting immune dysfunction and end-organ damage. Among these alterations, glucose metabolic reprogramming—characterized by a shift from oxidative phosphorylation(OXPHOS) to aerobic glycolysis, enhanced pentose phosphate pathway (PPP) activity, or a disrupted tricarboxylic acid (TCA) cycle—is particularly prominent in SLE (7). This phenomenon is observed in patient-derived monocytes/macrophages, neutrophils, DCs, T cells and B cells, where it drives aberrant activation, differentiation, and proinflammatory responses (6, 8–10). Within target organs such as the kidney, resident cells (e.g., mesangial and tubular epithelial cells) also exhibit similar metabolic alterations, directly contributing to local inflammation and tissue injury (11). However, a comprehensive synthesis of how glucose metabolic reprogramming operates specifically within the renal microenvironment to drive LN pathogenesis remains lacking.

Therefore, this review aims to: first, systematically elaborate the specific changes and functional consequences of glucose metabolic reprogramming in both infiltrating immune cells and renal resident cells within the context of LN; second, comprehensively evaluate potential therapeutic strategies targeting these metabolic pathways for LN treatment, including drug repurposing, novel small molecules, and combination approaches with biologics; and third, discuss future directions such as real-time metabolic imaging and personalized metabolic profiling to advance the era of precision medicine in LN/SLE. By focusing on the glucose metabolism axis within the kidney, we hope to provide an integrative framework for understanding LN pathogenesis and developing novel immunomodulatory approaches for this devastating complication of SLE.

## 2 Glucose metabolic reprogramming in innate immune cells

As the first line of defense and key drivers of inflammation in the pathogenesis of SLE, innate immune cells contribute profoundly to disease initiation and progression through their fundamental functions, including antigen presentation, release of inflammatory mediators, and immune complex clearance. Accumulating evidence now highlights that the functional aberrations of these cells are intimately linked to intrinsic glucose metabolic reprogramming, offering a novel perspective for understanding the immune dysregulation in SLE. Current investigations have primarily focused on monocytes/macrophages, DCs, and neutrophils, elucidating the critical role of their metabolic remodeling in SLE pathology. In contrast, the glucose metabolic characteristics and functional relevance of basophils, eosinophils, natural killer cells, and mast cells in SLE remain largely unexplored, representing a significant gap in our current knowledge.

### 2.1 Glucose metabolic reprogramming in monocytes/macrophages fuels kidney inflammation in lupus nephritis

The pathogenesis of SLE and its severe renal complication, LN, involves complex immune dysregulation. Macrophages, as key effector cells of the innate immune system, play multifaceted and central roles in this process, including immune complex clearance, antigen presentation, inflammation regulation, and tissue repair and fibrosis (12). Emerging research increasingly highlights that the functional state of macrophages is inextricably linked to their metabolic reprogramming, particularly the profound remodeling of glucose metabolic pathways. This bidirectional regulatory relationship is crucial in the initiation, progression, and tissue injury of LN.The classical (M1) and alternative (M2) activation states of macrophages are tightly coupled to their metabolic signatures. M1 macrophages, typically induced by pro-inflammatory signals such as interferon-gamma and lipopolysaccharide, rely heavily on enhanced glycolysis to rapidly generate ATP and support the massive synthesis of inflammatory mediators, while their OXPHOS and mitochondrial function are relatively suppressed. In contrast, M2 macrophages, associated with tissue repair and anti-inflammatory responses, depend more on OXPHOS and fatty acid oxidation (FAO) to meet their energy demands (13). In the context of LN, this metabolic polarization imbalance frequently skews towards the pro-inflammatory M1 phenotype. This is particularly evident within the renal microenvironment, where infiltrating macrophages in both the glomeruli and tubulointerstitium of LN patients and mouse models predominantly exhibit an M1-like phenotype, driving local inflammation and injury (14, 15).

The aberrant activation of the glycolytic pathway serves as a key engine driving the inflammatory response of macrophages in LN. Evidence from multiple levels supports this assertion. Firstly, upregulation of key glycolytic enzymes and transporters, along with increased glycolytic flux, is commonly observed in monocytes/macrophages from lupus-prone mouse models and SLE/LN patients (16). For instance, elevated expression of glucose transporter 1 (GLUT1) and hexokinase 2 (HK2) facilitates glucose uptake and the first rate-limiting step of glycolysis, providing ample metabolic substrates for M1 macrophage activation (17). Secondly, immune complexes (ICs), particularly IgG-containing ICs (IgG ICs), can potently induce glycolysis in macrophages via cross-linking of Fcγ receptors in a mammalian target of rapamycin (mTOR)-hypoxia-inducible factor-1α (HIF-1α)-dependent manner (18). Critically, the activation of this pathway is not merely an *in vitro* phenomenon; studies have confirmed the upregulation of HIF-1α and its target genes, such as pyruvate dehydrogenase kinase 1 (PDK1) and lactate dehydrogenase A (LDHA), specifically within the CD68+ macrophages infiltrating the kidneys of LN patients and lupus-prone mice (19, 20). PDK1 inhibits the activity of pyruvate dehydrogenase (PDH), preventing pyruvate entry into the TCA cycle and shunting it towards lactate production. This process not only boosts glycolytic flux but also promotes M1 polarization, as lactate itself can further reinforce this phenotype (19, 21). Functional evidence comes from interventional studies: the use of glycolytic inhibitors (e.g., 2-deoxyglucose) or targeting HIF-1α significantly reduces the release of pro-inflammatory cytokines (e.g., IL-1β) from IgG IC-stimulated macrophages and ameliorates renal inflammation and injury in mouse models of LN (18). Clinical observations also indicate that sodium-glucose cotransporter 2 (SGLT2) inhibitors like empagliflozin, by reducing glucose uptake and glycolysis in peri-tubular macrophages, can promote their shift towards an M2 phenotype and have shown potential renal protective effects in small-scale clinical studies involving LN patients (22, 23).

Complex positive feedback loops exist between glycolysis and innate immune signaling pathways, exacerbating the inflammatory storm in LN. A prominent example is pyruvate kinase M2 (PKM2). PKM2 is upregulated in macrophages from SLE patients. It not only functions as a key glycolytic enzyme catalyzing the conversion of phosphoenolpyruvate to pyruvate but can also translocate to the nucleus to regulate inflammatory gene expression (24, 25). Studies demonstrate that activation of Toll-like receptor (TLR) 4, 7, and 9 pathways can elevate PKM2 levels, while PKM2 overexpression, in turn, amplifies signaling through these TLR pathways, creating a self-reinforcing pro-inflammatory cycle (26). In lupus mouse models, treatment with PKM2 inhibitors effectively reduces renal immune complex deposition and improves nephritis (27). PKM2 also impairs cognition in SLE by promoting microglial synaptic pruning via the β-catenin signaling pathway (25). Furthermore, type I interferons (IFN-I), central cytokines in SLE, can induce metabolic reprogramming in monocytes/macrophages, concurrently enhancing both glycolysis and OXPHOS. IFN-α stimulation increases the activity of isocitrate dehydrogenase 2 in the TCA cycle, leading to elevated production of α-ketoglutarate (α-KG). As a cofactor for histone demethylases such as KDM6A/B, α-KG can alter the histone methylation status at interferon-stimulated gene promoters, thereby establishing a form of epigenetic memory akin to “trained immunity,” which primes cells for a more robust and sustained response to subsequent IFN challenges (28). This suggests that targeting such metabolic-epigenetic cross-talk nodes (e.g., KDM6A/B) may offer novel strategies to dampen excessive IFN responses.

The interferon response-associated transcription factor IRF5 regulates the expression of OXPHOS-related genes in monocytes and plasmacytoid dendritic cells (pDCs). Its deficiency reduces mitochondrial activity and suppresses inflammatory pathways (29). Additionally, the ubiquitin-specific protease 18, a negative regulator of IFN signaling, when deficient, enhances M1 polarization, glycolysis, and mitochondrial reactive oxygen species (mtROS) production, leading to the release of mitochondrial DNA (mtDNA) into the cytosol (30). Released mtDNA can actives cyclic GMP-AMP synthase (cGAS)/stimulator of interferon genes (STING) pathway and further amplifying IFN-I responses (31, 32). Thus, increased glycolysis is considered an upstream event triggering mtROS and mtDNA release, forming a vicious cycle of “metabolism-mitochondrial damage-innate immune activation.”Research also indicates that specific transcription factors integrate metabolic and inflammatory programming. CCAAT/enhancer-binding protein beta (CEBPB) is highly expressed in the renal tissues of LN patients and correlates with disease activity. CEBPB can directly transcriptionally activate the BZW1 gene. BZW1, in turn, promotes endoplasmic reticulum stress and glycolysis in macrophages by regulating the phosphorylation level of eukaryotic translation initiation factor 2 alpha (eIF2α), thereby driving LN progression. Knocking down either CEBPB or BZW1 significantly alleviates nephritis in lupus mice (33).

Activation of the PPP provides crucial metabolic support for macrophages in LN to maintain redox homeostasis and sustain pro-inflammatory functions (34). The PPP is a major branch of glucose metabolism, primarily generating reduced nicotinamide adenine dinucleotide phosphate (NADPH) and ribose-5-phosphate. NADPH is essential for maintaining intracellular antioxidant systems and also for reactive oxygen species (ROS) production. In LN, significantly enhanced PPP activity has been detected in monocytes/macrophages from patients and, importantly, in CD68^+^ macrophages infiltrating infiltrating the kidneys, where they face high levels of oxidative stress and relative hypoxia (35). This metabolic adaptation equips these infiltrating M1 macrophages with the capacity to counteract oxidative stress, thereby stabilizing their inflammatory state and enabling their survival and function in the hostile renal milieu. Furthermore, its metabolites may also directly regulate inflammatory gene expression by influencing the uridine diphosphate glucose (UDPG)-P2Y14 receptor signaling pathway. Disruption of the PPP leads to downregulation of M1 macrophage markers (e.g., iNOS, TNF-α, IL-6) (36). Intriguingly, PPP activity is also linked to the immune tolerance function of macrophages. Studies show that the clearance of apoptotic cells reduces the expression of PPP-related genes in macrophages. Artificially enhancing PPP activity impairs macrophage phagocytosis of apoptotic cells and exacerbates inflammatory responses during efferocytosis, worsening lupus-like symptoms in SLE models (34). This suggests that fine-tuning PPP activity is critical for maintaining macrophage tolerance during the clearance of self-antigens, and its dysregulation may contribute to the perpetuation of autoimmunity.

The TCA cycle, essential for ATP production and biosynthetic precursors, is dysregulated in macrophages from patients with SLE, contributing to a pro-inflammatory phenotype (37). In M1 macrophages of SLE patients, the TCA cycle is fragmented, characterized by reduced citrate and elevated succinate levels (38). Succinate accumulation stabilizes HIF-1α, thereby promoting glycolysis and enhancing pro-inflammatory cytokine production. In contrast, M2 macrophages exhibit enhanced TCA cycle activity, with increased expression of citrate synthase and succinate dehydrogenase. TCA cycle intermediates, such as itaconate, play immunomodulatory roles by inhibiting succinate dehydrogenase, which reduces ROS production and supports M2 polarization (39). Given that M2 macrophages depend on OXPHOS for ATP generation, an intact and functional TCA cycle is critical for maintaining their differentiation. Inhibition of fumarate hydratase, a TCA cycle enzyme, leads to fumarate accumulation, disrupts the cycle, and suppresses M2 polarization (40). M2 polarization is driven by the IL-4/STAT6/PPARγ axis, which transcriptionally upregulates genes related to OXPHOS and FAO, thereby establishing the characteristic metabolic program of M2 and supporting its anti-inflammatory function (41–43). Clinically, reduced FH expression in whole blood samples from SLE patients suggests impaired M2 macrophage polarization in this disease. Citrate/isocitrate can be exported from mitochondria via the citrate carrier, regulating HIF-1α-dependent genes and stimulating glycolysis (39). HIF-1α also upregulates immune-responsive gene 1, promoting citrate conversion to itaconate. Itaconate accumulation inhibits succinate dehydrogenase, leading to succinate buildup, which further activates HIF-1α and glycolytic genes, sustaining the M1 phenotype( (44, 45). This metabolic remodeling, particularly the accumulation of succinate and itaconate, is not only a marker of inflammation but actively shapes the ability of infiltrating kidney macrophages to adapt to the local microenvironment, reinforcing their pro-inflammatory functions and contributing to tissue damage. Inhibiting CIC suppresses glycolysis, blocks the Irg1/itaconate pathway, restores normal mitochondrial cycling, and reduces citrate and itaconate accumulation, thereby enhancing OXPHOS flux and facilitating the M1-to-M2 phenotypic switch (39, 44, 46). However, the role of itaconate in inflammation is complex and context-dependent. Contrary to a purely pro-inflammatory role, emerging evidence suggests itaconate can also exert potent anti-inflammatory and immunoregulatory effects by activating the anti-inflammatory transcription factor Nrf2 and inhibiting the NLRP3 inflammasome (47, 48). In the context of SLE, itaconate has been reported to be protective (49). This duality underscores that the net effect of itaconate likely depends on its concentration, the cellular context, and the specific phase of the inflammatory response. Further research is needed to dissect its precise role in the pathogenesis of lupus nephritis.

**Figure 1:**
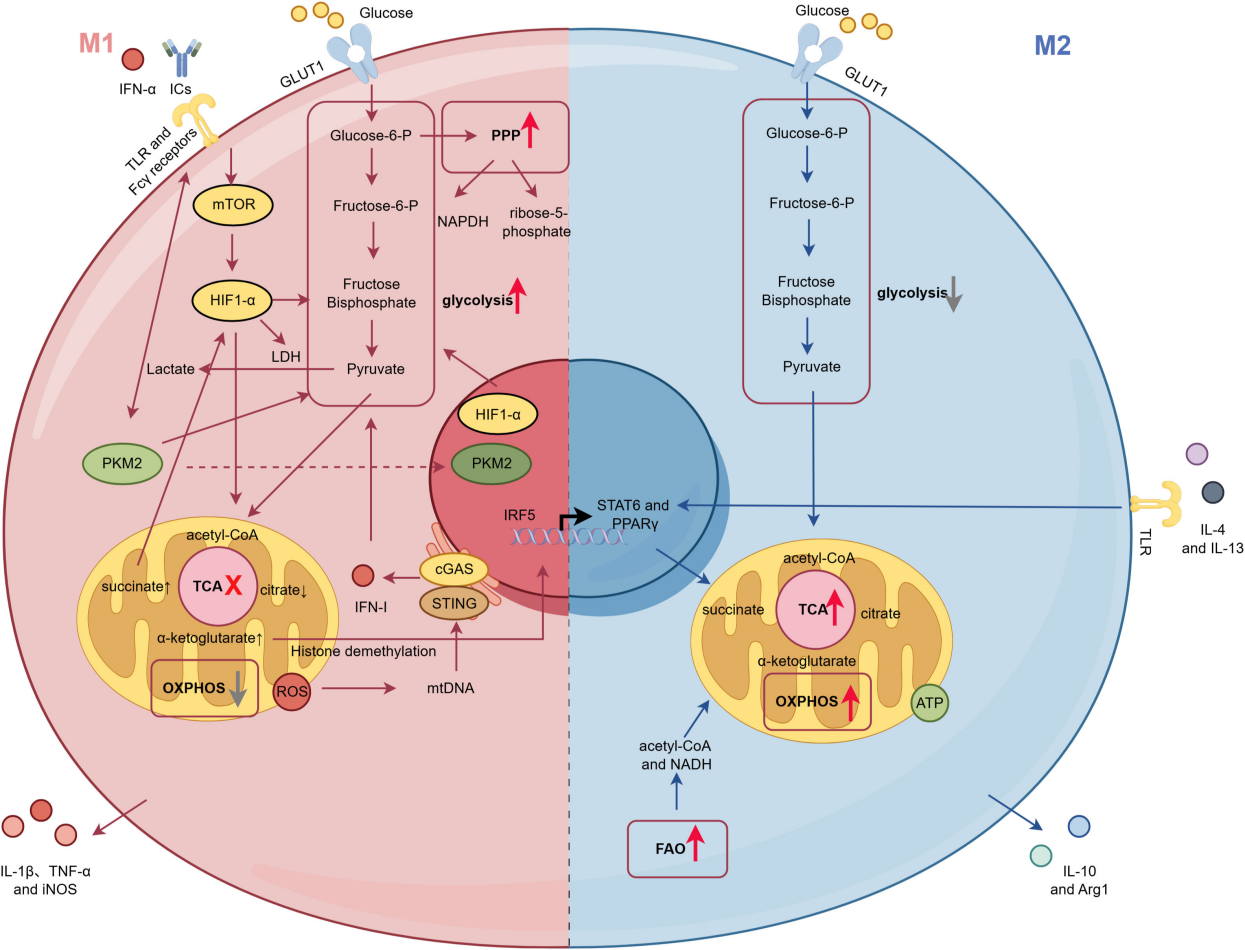
Metabolic reprogramming of kidney-infiltrating macrophages in lupus nephritis. Within the inflamed kidney microenvironment of lupus nephritis (LN), signals such as IFN-I and ICs drive macrophage/monocyte polarization toward a pro-inflammatory M1 phenotype by activating pathways including the mTOR and IRF5. Central to this process is HIF-1α-dependent metabolic reprogramming. M1 Phenotype (Left): Characterized by markedly enhanced glycolysis and PPP flux. Glucose is avidly taken up via GLUT1 and converted to lactate. The glycolytic enzyme PKM2 dimerizes and translocates to the nucleus to participate in gene regulation. The TCA cycle is fragmented, leading to succinate accumulation, which stabilizes HIF-1α, creating a positive feedback loop. Mitochondrial OXPHOS is dysfunctional, increasing RO) production and causing mtDNA release into the cytosol. This activates the cGAS-STING pathway, amplifying the IFN-I response and establishing a vicious cycle of “metabolism-mitochondrial damage-inflammation.” This metabolic state supports the production of pro-inflammatory mediators such as IL-1β, TNF-α, and iNOS. M2 Phenotype (Right): Its classical polarization is driven by the IL-4/STAT6/PPARγ signaling axis. The core transcription factors STAT6 and PPARγ transcriptionally upregulate genes involved in FAO and OXPHOS, thereby establishing the characteristic M2 metabolic program featuring an intact TCA cycle, efficient OXPHOS, and active FAO, coupled with moderate glycolysis to support high-yield ATP production. This metabolic state supports the production of anti-inflammatory factors like IL-10 and Arg1, facilitating immune regulation and tissue repair. However, M2 polarization and function are relatively impaired in SLE.

In summary, multiple nodes of glucose metabolism are intricately intertwined with factors such as HIF-1α, PKM2, IFN signaling, TLR pathways, and epigenetic modifications, forming a complex regulatory network that collectively determines the polarization fate and inflammatory output of macrophages within the renal microenvironment of LN (**Figure 1**). Targeting these key metabolic enzymes (e.g., HK2, PKM2), transporters (e.g., GLUT1), or regulatory factors (e.g., HIF-1α, PPP) to “re-educate” macrophages—shifting them from a pro-inflammatory M1 phenotype to a reparative M2 phenotype or directly inhibiting their excessive inflammatory functions—offers a highly promising strategy for developing novel immunometabolic therapies for LN. Future research should focus on validating these metabolic targets in human LN samples and exploring how to achieve precise and effective metabolic interventions within the complex *in vivo* microenvironment.

### 2.2 Immunometabolic dysregulation of neutrophils in SLE pathogenesis

In SLE, neutrophils primarily exacerbate autoimmune responses by releasing neutrophil extracellular traps (NETs), which promote autoantigen exposure, interferon production, and end-organ damage (50). Neutrophil dysfunction is associated with significant metabolic reprogramming, though its specific metabolic profile remains incompletely defined. Studies indicate that SLE neutrophils exhibit alterations in ROS production, mitochondrial function, and energy metabolism pathways. On one hand, enhanced glycolysis and PPP activity provide the metabolic foundation for NADPH-dependent ROS bursts, which are closely linked to NETosis, a specialized form of cell death characterized by the release of immunogenic NETs and DNA (51). On the other hand, mitochondrial dysfunction is particularly prominent: ribonucleoprotein ICs can induce mitochondrial membrane depolarization and accumulation of mitochondrial ROS, promoting the release of oxidized mtDNA and thereby driving a robust type I interferon response. Notably, mtROS scavenging has been shown to reduce disease activity in lupus mouse models, highlighting its critical role in immunometabolic crosstalk (52, 53). Furthermore, the mTOR signaling pathway regulates ATP production and calcium homeostasis, influencing neutrophil chemotaxis and activation, while its inhibition leads to impaired function (54). Although current evidence is largely derived from associative studies or model systems, whether a consistent metabolic reprogramming phenotype exists in SLE neutrophils remains unclear. Future studies should employ metabolomics and single-cell technologies to systematically characterize the metabolic networks in patient-derived neutrophils, providing a foundation for targeting metabolic pathways in SLE therapy.

### 2.3 Dendritic cells metabolism in lupus nephritis: Insights from other models and the need for confirmation

In autoimmune disease, such as SLE, rheumatoid arthritis (RA) and psoriasis, DCs act as pivotal antigen-presenting cells that aberrantly activate autoreactive T and B cells while producing copious pro-inflammatory cytokines, particularly type I interferons, thereby driving and sustaining systemic autoimmunity (55). Both myeloid dendritic cells (mDCs) and plasmacytoid dendritic cells (pDCs) are implicated in SLE pathogenesis, and emerging evidence from other inflammatory contexts suggests they may undergo significant metabolic reprogramming, though direct confirmation in SLE/LN remains limited.

In mDCs, studies in other disease models and *in vitro* systems have characterized a metabolic state shift from a catabolic profile under resting conditions—reliant on OXPHOS, FAO, and glutaminolysis—toward an anabolic state upon activation (56). Glycolysis is the main rapid energy source that pathogenic dendritic cells preferentially utilize. In the context of autoimmune diseases such as SLE and RA, this transition is primarily driven by abnormal ICs through crosstalk between Fcγ receptors (FcγRs) and TLRs, and depends on transcriptional activation mediated by interferon regulatory factor 5 (IRF5) (57). This IRF5 activation not only upregulates pro−inflammatory cytokine gene transcription but also cooperatively induces glycolytic reprogramming, thereby amplifying TNF production. Key signaling pathways such as mTOR, HIF−1α, and the TBK1/IKKϵ–Akt axis are activated, collectively promoting a marked increase in glycolytic flux (57–60). This metabolic shift not only rapidly supplies energy but also enhances flux through the PPP to support nucleotide synthesis and NADPH−dependent ROS production, further amplifying inflammatory signaling (61). Concurrently, glycolytic intermediates are channeled into *de novo* fatty acid synthesis to support organelle biogenesis and massive protein secretion, meeting the enhanced demands for antigen presentation and pro−inflammatory mediator release (60). Notably, sustained mTORC1 activation, while accelerating mDC maturation, also leads to excessive ROS accumulation, which may impair cellular function and exacerbate the inflammatory microenvironment.

The metabolic regulation of pDCs in SLE is complex and, in some aspects, paradoxical. Studies in TLR9-activated pDCs have shown that they require enhanced FAO and OXPHOS via an autocrine IFN-I receptor-dependent pathway to support robust IFN-I production under physiological conditions (62). However, in SLE patients, the interferon−producing capacity of pDCs is significantly diminished, and their metabolic adaptability appears dysregulated. Transcriptomic analyses of pDCs from SLE patients have revealed an altered expression of genes involved in cellular senescence, autophagy (e.g., ATG14), and lysosomal protein degradation, suggesting that their metabolic homeostasis may be compromised (63). Furthermore, studies in systemic sclerosis and SLE suggest that suppression of the unfolded protein response may disrupt the diversion of glycolytic intermediates into the serine synthesis pathway, thereby breaking metabolic checkpoints and potentially promoting pDC over−activation (64). Although the direct causal relationship between metabolic alterations and pDC functional defects remains unclear, metabolic dysregulation likely exacerbates the loss of their immunoregulatory functions and promotes persistent autoantigen presentation and aberrant interferon pathway activation.

Furthermore, the role of key glycolytic enzymes in DCs may differ from that observed in other immune cells. For instance, studies in LPS-activated DCs have shown that PKM2 transitions from an active tetrameric form to an inactive monomer, a shift associated with increased glycolysis, contrasting with the nuclear translocation of dimeric PKM2 observed in T cells and macrophages (65). This highlights the cell-type specificity of metabolic regulation and underscores the need for direct investigation in DCs within the context of SLE.

As another essential component of glucose catabolism, OXPHOS plays a pivotal role in regulating DCs functions. Emerging evidence indicates that OXPHOS promotes the acquisition of tolerogenic properties in DCs by suppressing their maturation (66). Mechanistically, enhanced OXPHOS activity in DCs leads to markedly reduced production of pro-inflammatory cytokines, including IL-7, IL-8, and IL-1β, while facilitating the differentiation of regulatory T cells (Tregs). This regulatory mechanism presents a promising therapeutic avenue for various autoimmune diseases, such as RA and type 1 diabetes (67, 68).

In summary, based on studies in other inflammatory contexts and *in vitro* models, mDCs and pDCs in SLE are hypothesized to contribute to the breakdown of immune tolerance and amplification of inflammatory cycles through distinct metabolic mechanisms. mDCs may do so via a glycolytic−biased anabolic activation driven by IRF5-dependent transcriptional programs, and pDCs potentially via functional impairment linked to disrupted metabolic adaptation and UPR dysregulation. However, direct experimental evidence confirming these precise metabolic mechanisms in patient-derived renal dendritic cells or within the kidneys of LN models remains limited and warrants future investigation. Future studies should further elucidate the precise regulatory networks of metabolic reprogramming in different DC subsets and their specific contributions to SLE/LN pathogenesis, providing a theoretical foundation for developing metabolism−targeted immunomodulatory strategies.

## 3 Glucose metabolic reprogramming in adaptive immune cells

### 3.1 Pathogenic T cell subsets in lupus nephritis are fueled by glycolytic reprogramming

T cells play a central pathogenic role in SLE and LN, where dysregulated hyperactivity of helper T cell subsets promotes autoantibody production and tissue injury, while impaired regulatory T cell (Treg) function leads to a loss of immune tolerance, collectively exacerbating systemic autoimmunity (69). Emerging evidence positions metabolic reprogramming of T lymphocytes not as an epiphenomenon but as a cardinal driver of their aberrant activation, differentiation, and effector functions. Pathogenic CD4+ T cell subsets, notably follicular helper T (Tfh) and T helper 17 (Th17) cells, exhibit a profound metabolic rewiring characterized by a state of generalized metabolic hyperactivity (70). In the context of LN, these metabolically hyperactive T cell subsets are key drivers of renal injury. Tfh cells, expanded in the peripheral blood and secondary lymphoid organs, are indispensable for the generation of pathogenic autoantibodies that form nephritogenic immune complexes deposited in the glomeruli (71). Concurrently, Th17 cells and their signature cytokine IL-17 are found infiltrating the kidneys of LN patients, where they promote local inflammation and contribute to tubulointerstitial damage (72).

Naive CD4+ and CD8+ T cells primarily utilize OXPHOS to meet bioenergetic demands (73, 74). Upon chronic or dysregulated stimulation in SLE, T cells undergo a metabolic switch towards aerobic glycolysis, a phenomenon orchestrated by coordinated signaling and transcriptional networks (75–77). Upregulation of the glucose transporter GLUT1 via the PI3K-AKT-mTORC1 axis facilitates increased glucose uptake (77, 78). Downstream, master transcriptional regulators including c-Myc, HIF-1α, and estrogen-related receptor α are activated, collectively inducing the expression of rate-limiting glycolytic enzymes such as HK2 and PKM2 (71–74, 79). This metabolic hyperactivity is a hallmark of SLE T cells, which exhibit concurrently elevated glycolysis and OXPHOS, a state of “dual metabolic hyperactivity” that fuels their pathogenic functions (76, 80). HIF-1α-mediated promotion of glycolysis is a key metabolic switch supporting the differentiation and function of multiple T cell lineages, including Th1, Th17, Tfh, and CD8+ T cells, though its importance in Th2 differentiation is not fully established (81).

This glycolytic shift is particularly critical for the ontogeny and function of key pathogenic T cell subsets. First, Tfh cells, whose expansion strongly correlates with SLE disease activity, demonstrate a unique metabolic dependency. Tfh cells from lupus-prone mice and SLE patients exhibit a heightened reliance on glycolysis compared to their counterparts induced by foreign antigens (82, 83). This selective metabolic vulnerability directly links to renal pathology. Pharmacological inhibition of glycolysis with 2-deoxy-D-glucose (2-DG) or allosteric activation of PKM2 tetramerization with TEPP-46 selectively abrogates the expansion of autoreactive Tfh cells, germinal center responses, and autoantibody production in murine lupus models, while sparing antigen-specific immune responses (71, 84). Critically, this reduction in systemic autoimmunity translates to renal protection, as evidenced by decreased immune complex deposition and ameliorated nephritis in treated mice. The mechanism extends beyond bioenergetics: dimeric PKM2 can translocate to the nucleus and function as a transcriptional coactivator. TEPP-46-mediated tetramerization sequesters PKM2 in the cytosol, reducing its nuclear availability and subsequent coactivation of the Tfh lineage-defining transcription factor Bcl-6, thereby impairing Tfh differentiation at a transcriptional leve (72, 84, 85–87). Furthermore, calcium/calmodulin-dependent protein kinase IV (CaMK4), overexpressed in T cells from active SLE patients, promotes glycolysis by enhancing GLUT1 expression, directly fueling the production of Th17 and Tfh-associated cytokines (88). In line with this, inhibiting CaMK4 has been shown to reduce GLUT1 expression and IL-17 production, highlighting a potential therapeutic axis (88).

Second, glycolytic reprogramming underpins the Th17/Treg imbalance. Th17 cell differentiation is intrinsically linked to high glycolytic flux, with key transcription factors like RORC expression and signaling pathways (e.g., mTORC1) being both regulators and beneficiaries of glycolysis (89, 90). HIF-1α, stabilized in Th17 cells, induces glycolytic genes expression (8, 91). In stark contrast, the differentiation and suppressive function of Treg cells are supported by FAO and OXPHOS (92–94). Foxp3 represses c-Myc, thereby constraining glycolysis (95, 96). In SLE, the overarching glycolytic metabolic milieu preferentially drives Th17 differentiation while simultaneously impairing Treg generation and function, creating a self-reinforcing pro-inflammatory bias (78, 97–99). This imbalance is not merely systemic; it is operative within the inflamed kidney, where the balance between pro-inflammatory Th17 and protective Tregs contributes to the severity of renal injury (82). This metabolic preference is also evident within circulating Tfh subsets, displays the highest glycolytic activity and a pronounced glucose dependency for its function (82, 100, 101). Evidence also suggests that following systemic infection, memory CD8+ T cells may utilize serum acetate accumulation to enhance glycolytic activity, thereby contributing to disease pathogenesis (102).

The hyperactive glycolytic flux in SLE T cells is coupled with a parallel dysregulation of the PPP, a critical branching pathway from glycolysis. The oxidative arm of the PPP generates NADPH, the primary cellular reductant for combating oxidative stress and for *de novo* lipid synthesis, and ribose-5-phosphate, an essential precursor for nucleotide biosynthesis (103).

In SLE patients and lupus T cells, the oxidative PPP is aberrantly activated (71, 104, 105). Transcriptomic and metabolomic profiling of Tfh cells from lupus-prone mice reveals upregulation of oxidative PPP-related genes (71). This activation serves dual, potentially interconnected, purposes: First, to mitigate profound oxidative stress. SLE T cells exist in a state of chronic activation with dysfunctional mitochondria, leading to excessive mtROS production. Upregulation of the PPP generates NADPH to regenerate reduced glutathione, enabling cell survival under intense redox pressure (106, 107). Second, to fuel rampant biosynthesis. The clonal expansion of autoreactive T and B cell clones necessitates massive nucleotide synthesis for DNA/RNA replication. The ribose-5-phosphate produced by the PPP is indispensable for meeting this biosynthetic demand (103). Notably, treatment with 2-DG might ameliorates the overactive PPP in lupus T cells, suggesting that glycolytic inhibition may have pleiotropic effects on interconnected metabolic networks (71). Intriguingly, PPP metabolites like gluconolactone have been reported to modulate the Treg/Th17 balance, indicating that PPP flux can directly influence T cell fate decisions (108).

Contrary to the classic Warburg effect observed in some cancers, the upregulation of glycolysis in SLE T cells is not accompanied by a suppression of mitochondrial metabolism (76, 83). Instead, a state of “dual metabolic hyperactivity” exists, characterized by concurrently elevated glycolysis and OXPHOS. This paradox implies a profound rerouting of TCA cycle intermediates.

Lupus T cells exhibit multiple mitochondrial abnormalities, including membrane hyperpolarization and electron transport chain inefficiencies, yet demonstrate increased oxygen consumption rates (8, 109). This likely reflects compensatory but inefficient respiration, further exacerbating mtROS production (110). Concurrently, the TCA cycle undergoes significant metabolic rewiring: Rapidly proliferating cells require constant replenishment of TCA cycle intermediates (e.g., oxaloacetate, α-KG) that are siphoned off as precursors for amino acid and nucleotide synthesis. Lupus Tfh cells display enhanced glutaminolysis and malate-aspartate shuttle activity, key pathways for importing non-glucose carbons into the TCA cycle (78). Citrate from the TCA cycle is exported to the cytosol and cleaved into acetyl-CoA, a critical building block for *de novo* fatty acid and cholesterol synthesis required for membrane biogenesis in rapidly expanding cells. TCA cycle intermediates (e.g., succinate, fumarate) and derivative metabolites like acetyl-CoA can function as signaling molecules. They influence histone acetylation, DNA methylation and stabilize transcription factors HIF-1α, thereby transcriptionally reinforcing the pathogenic T cell program. For instance, acetyl-CoA derived from citrate may fuel histone acetylation at pro-inflammatory gene loci, perpetuating the autoreactive state (106). Itaconate and α-KG, two key TCA cycle intermediates with immunomodulatory properties, may contribute to SLE pathogenesis by metabolically reprogramming CD4+T cells, thereby influencing the critical balance between pro-inflammatory TH17 and regulatory Treg cell differentiation (111).

**Figure 2:**
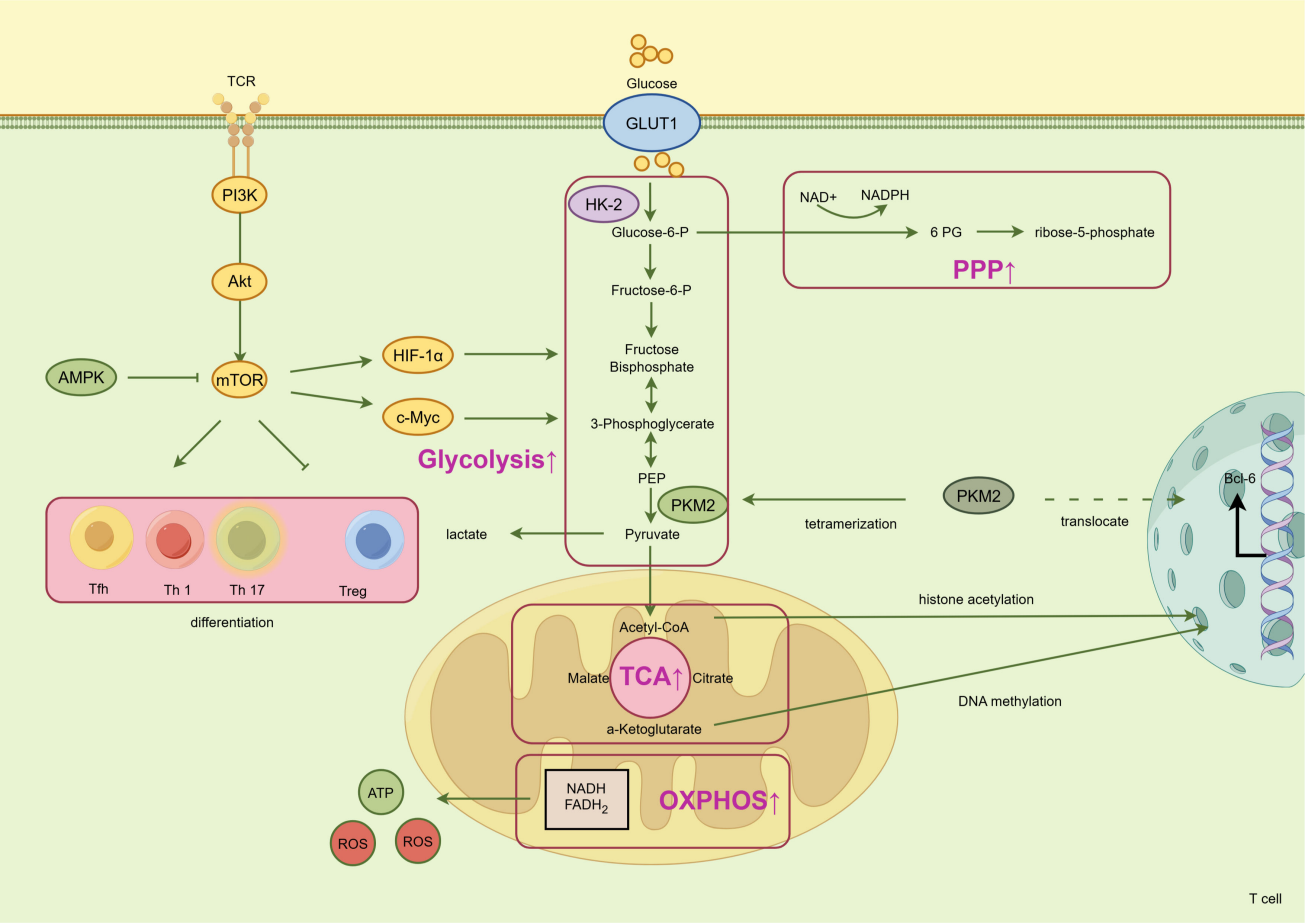
Glycolysis-centric metabolic reprogramming in pathogenic CD4^+^T cells drives renal injury in LN. In LN, chronic or dysregulated TCR signaling activates the mechanistic target of mTORC1 via the PI3K-AKT pathway. Serving as a central hub, mTORC1 orchestrates a series of metabolic alterations. Its downstream transcription factors, HIF-1α and c-Myc, upregulate the expression of the glucose transporter GLUT1 and key glycolytic enzymes (e.g., HK2, PKM2), driving enhanced aerobic glycolysis. Glucose metabolism is also diverted into the pentose phosphate pathway, generating NADPH to combat oxidative stress and providing ribose-5-phosphate for nucleotide synthesis. The glycolytic product pyruvate is converted to acetyl-CoA, which fuels the TCA cycle. This hyperactive TCA cycle is supported by anaplerotic reactions like glutaminolysis to replenish biosynthetic precursors. However, mitochondrial function is aberrant, characterized by increased yet inefficient oxidative phosphorylation, leading to excessive production of reactive oxygen species. Metabolic intermediates serve signaling functions: acetyl-CoA acts as a substrate for histone acetylation; α-KG influences DNA methylation; and the dimeric form of PKM2 can translocate to the nucleus to co-activate Bcl-6, a key transcription factor forTfh cells. AMPK, an energy sensor, inhibits mTOR activity. Collectively, this glycolysis-centric metabolic reprogramming, coupled with mitochondrial dysfunction and oxidative stress, creates a metabolic milieu that promotes the differentiation of inflammatory Th17 and Tfh cells while impairing the function of Tregs, thereby breaking immune tolerance, promotes the formation of autoantibodies that deposit in the kidney, and potentially leads to the infiltration of inflammatory cells into the renal tissue, ultimately driving glomerulonephritis and tubulointerstitial fibrosis.

In conclusion, metabolic reprogramming of T cells in SLE constitutes a multilayered, interconnected pathological network. Aberrantly enhanced aerobic glycolysis sits at its core, coupled with PPP activation to manage redox stress and biosynthetic needs, and linked to a dysfunctional yet hyperactive TCA cycle that supports anaplerosis and provides signaling metabolites. This network is orchestrated by master regulators like mTOR, adenosine Monophosphate-Activated Protein Kinase (AMPK), and CaMK4, culminating in the expansion of pathogenic Tfh and Th17 cells and the impairment of Tregs, thereby dismantling immune tolerance (**Figure 2**). The pathological consequences of this systemic metabolic dysregulation converge on the kidney, driving the production of nephritogenic autoantibodies and fostering a local inflammatory milieu that perpetuates LN. Therapeutic strategies targeting this metabolic vulnerability, ranging from direct enzyme inhibition to modulation of upstream signals, have demonstrated compelling efficacy in preclinical models. The translational challenge ahead lies in safely harnessing these metabolic modulators in the clinic and developing precision medicine approaches based on individual patient metabolic signatures. Glucose metabolic reprogramming of dysregulated immune cells stands as a promising and potentially paradigm-shifting frontier in the treatment of SLE.

### 3.2 B cell metabolism supports autoantibody production and intrarenal pathogenesis in lupus nephritis

B cells drive SLE pathogenesis by differentiating into autoreactive plasma cells that produce pathogenic autoantibodies, acting as antigen-presenting cells to activate autoreactive T cells, and secreting pro-inflammatory cytokines, thereby collectively promoting immune complex deposition, end-organ damage, and systemic autoimmunity (112). The ultimate consequence of this B cell dysregulation is the production of high-affinity pathogenic autoantibodies, particularly anti-dsDNA antibodies, whose deposition in the glomeruli is the pathological hallmark of LN (113). The pro-inflammatory milieu of SLE drives profound glycolytic rewiring in B lymphocytes, facilitating their aberrant activation, proliferation, and effector functions. This metabolic shift exhibits remarkable subset-specific features governed by discrete molecular machinery (114).

Naïve B cells in SLE undergo significant glucose metabolic reprogramming. Compared to healthy individuals, naïve B cells from SLE patients exhibit enhanced glycolytic activity, characterized by upregulation of key glycolytic enzymes such as GAPDH (115). This metabolic shift is primarily driven by TLR9 activation and IL-21 signaling, and correlates with disease activity (116, 117). Although resting naïve B cells normally rely on fatty acid oxidation for energy, within the inflammatory milieu of SLE, their metabolic program shifts toward glycolysis early on to meet the energetic and biosynthetic demands of aberrant activation, thereby contributing to the initiation and perpetuation of autoimmune responses (118).

**Figure 3:**
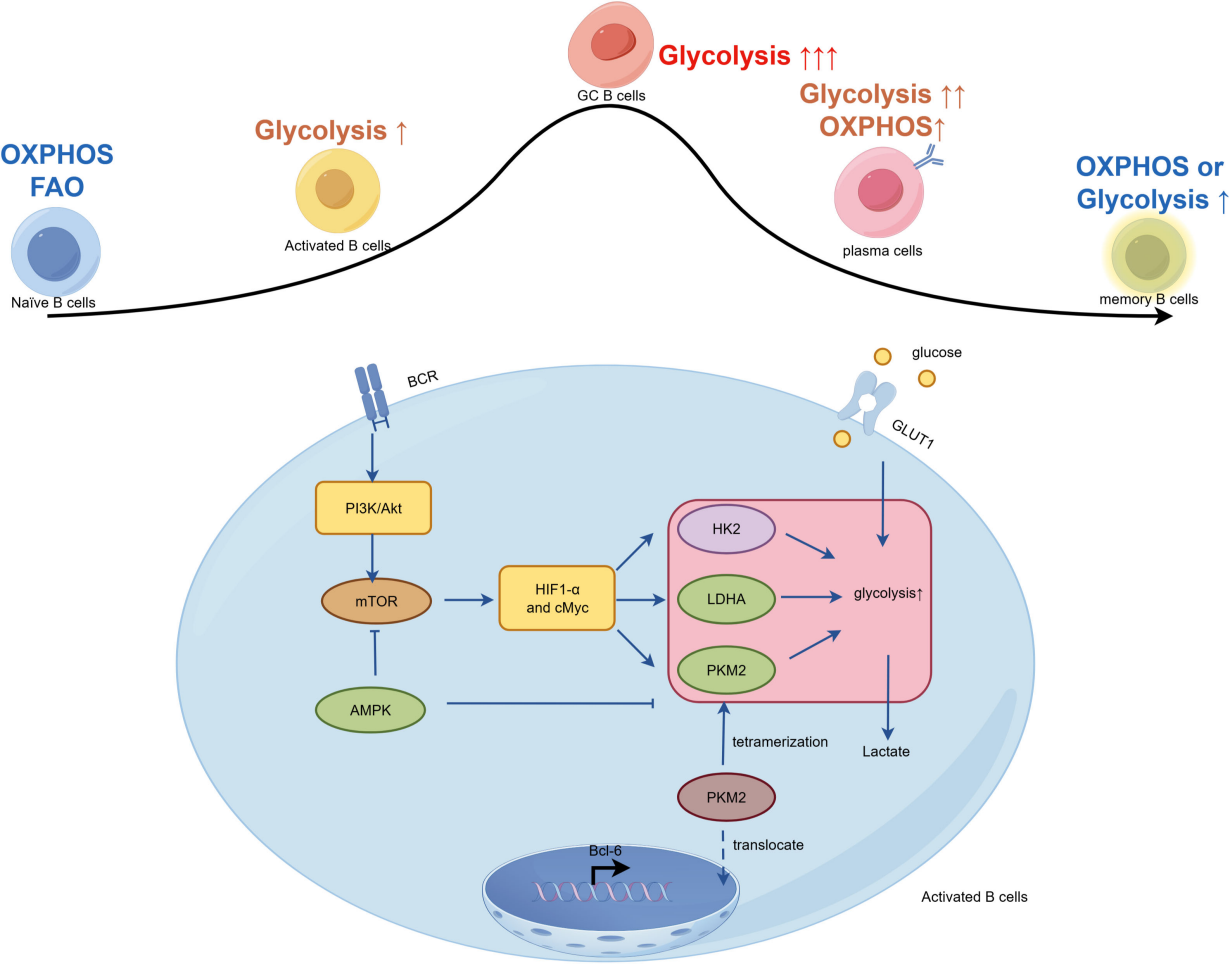
Metabolic reprogramming during B cell differentiation contributes to autoantibody production in LN. B cells undergo distinct metabolic reprogramming at different differentiation stages in SLE. Upon activation by autoantigens and inflammatory signals, B cells switch from OXPHOS and FAO to a glycolytic state. This metabolic shift is driven by the PI3K/Akt/mTORC1 signaling axis and downstream transcription factors HIF-1α and c-Myc, which upregulate glucose uptake and glycolytic enzymes. GC B cells exhibit an extreme dependence on glycolysis to support rapid proliferation and antibody affinity maturation. Differentiated plasma cells utilize both enhanced glycolysis and OXPHOS to sustain high-rate antibody synthesis and secretion, while memory B cells maintain metabolic flexibility. Specialized B-cell subsets such as ABCs and Bregs, which also undergo distinct glycolytic reprogramming in SLE, are not depicted in this simplified schema. The ultimate outcome of this metabolic support is the sustained production of high-affinity, pathogenic autoantibodies that form immune complexes in the glomeruli.

B-cell receptor (BCR) engagement triggers robust activation of the PI3K-AKT pathway, stabilizing c-Myc and HIF-1α (115, 119). HIF-1α transcriptionally induces a full suite of glycolytic enzymes, including HK2, PKM2, and LDHA (27, 120). The role of PKM2 is particularly critical. In its low-activity dimeric form, PKM2 translocates to the nucleus where it functions as a transcriptional coactivator, interacting with factors such as Bcl-6 to potentiate B cell activation and Tfh cell differentiation. In SLE patients and lupus-prone mice, PKM2 expression is markedly elevated in activated B cells and Tfh cells. Pharmacological promotion of PKM2 tetramerization with TEPP-46 sequesters the enzyme in the cytoplasm, suppresses glycolysis and nuclear signaling, and ameliorates disease in MRL/lpr mice by reducing Tfh cell expansion and autoantibody production (72). Concurrently, the AMPK/mTOR regulatory balance is disrupted, with heightened mTORC1 activity and suppressed AMPK signaling further amplifying glycolytic flux. Recent studies have shown that the traditional Chinese medicine formula Jieduquyuziyin prescription inhibits B-cell proliferation and activation by regulating metabolic reprogramming through the AMPK/PKM2 signaling pathway, thereby ameliorating damage in MRL/lpr mice (**Figure 3**) (121, 122).

Activated B cells further differentiate into germinal center B cells (GC B cells), which are central to the production of pathogenic autoantibodies in LN. These cells exhibit significant glucose metabolic reprogramming. Compared to early-activated B cells, GC B cells exhibit markedly enhanced glucose utilization and mitochondrial activity. They critically depend on glycolysis to fuel rapid clonal expansion, somatic hypermutation, and affinity maturation, which are essential for pathogenic autoantibody production in both murine models and SLE patients (123–125). This heightened glycolytic flux is often driven by mTORC1 signaling and further promoted by HIF-1α stabilization within the hypoxic germinal center microenvironment (115). Notably, autoreactive GC B cells in lupus show exceptional reliance on glucose oxidation, rendering them highly vulnerable to glycolytic inhibitors such as 2-DG; inhibiting glycolysis selectively reduces these pathogenic cells (126, 127). In the context of LN, it is noteworthy that ectopic lymphoid structures resembling germinal centers have been identified within the kidneys of patients with severe LN. The metabolic demands of infiltrating B cells within this intrarenal niche—characterized by hypoxia and nutrient limitation—are likely met by the same glycolytic reprogramming observed in secondary lymphoid organs, enabling local autoantibody production and contributing directly to tissue injury (125, 126). However, metabolic heterogeneity exists: glycolytic activation post-BCR stimulation may occur independently of mTORC1, and some subsets may adapt to fatty acid oxidation (128). In summary, the metabolic shift towards glycolysis is a central feature enabling GC B cells to acquire effector functions, produce autoantibodies, and drive SLE pathogenesis, highlighting their potential as targets for metabolic intervention.

As the terminal effectors of antibody secretion, plasma cells exhibit distinct metabolic reprogramming. Unlike activated B cells that primarily rely on aerobic glycolysis, differentiation into plasma cells shifts towards a mixed dependence on both glycolysis and OXPHOS to meet the enormous biosynthetic demands (129). Long-lived plasma cells (LLPCs), in particular, demonstrate significantly higher glucose uptake than their short-lived counterparts (SLPCs), as glucose is critical for their sustained survival and antibody glycosylation (130, 131). This process is regulated by key metabolic nodes: PKM2-mediated reprogramming is essential for B cell activation and plasma cell differentiation; heightened mTORC1 signaling drives plasmablast differentiation and steers metabolism towards glycolysis, whereas AMPK activation inhibits this process (10, 106, 132). In autoimmunity, such as in SLE, observed overactivation of mTORC1 in patient B cells promotes aberrant plasmablast differentiation and autoantibody production, highlighting the therapeutic potential of targeting this metabolic axis (124, 133).Within the inflamed kidney, antibody-secreting cells, including SLPCs and potentially LLPCs, can accumulate and sustain local autoantibody production. Their survival and function within this niche are likely underpinned by the same metabolic programs, particularly glucose metabolism, making them vulnerable to metabolic interventions.

Limited evidence is available regarding the metabolic regulation of memory B cells in SLE. One study comparing B cells from SLE patients and healthy donors reported that memory B cells (CD19+CD27+) from SLE patients exhibit enhanced metabolic capacity, characterized by elevated glycolytic activity and an altered balance between glycolysis and OXPHOS compared to healthy controls (10, 114). This observation suggests that metabolic dysregulation in SLE extends to the memory B cell compartment and may contribute to persistent autoimmune responses. However, the precise regulatory mechanisms underlying this metabolic reprogramming, its functional consequences for memory B cell activation and differentiation upon autoantigen re-exposure, and its specific role in SLE progression remain largely unexplored and require further investigation using primary cellular materials and rigorous experimental models.

In addition, two specialized B-cell subsets, age-associated B cells (ABCs; CD11c^+^T-bet^+^) and regulatory B cells (Breg), exhibit distinct glycolytic reprogramming that is integral to their pathogenic functions in SLE. ABCs, key pathogenic autoantibody producers, display a hypermetabolic state with markedly enhanced glycolytic capacity (134). This reprogramming is driven by IFN-γ signaling and executed via the downstream transcription factor T-bet, which upregulates the glycolytic gene program (113). Pharmacological inhibition of glycolysis 2-DG impairs ABCs formation, survival, terminal differentiation, and ameliorates autoimmunity *in vivo* (113). Conversely, IL-10^+^ Breg cells in SLE patients exhibit pro-inflammatory features, and their polarization and function critically depend on glycolysis rather than OXPHOS. This metabolic preference is driven by the MAPK/ERK/p38-c-Myc axis, which upregulates glycolytic enzymes,such as HK2, PFKFB3, LDHA, and lactate production (135). Inhibiting this pathway or directly blocking glycolysis ablates their pathogenic capacity to drive inflammatory T-helper responses (136).

B lymphocytes in SLE undergo comprehensive metabolic reprogramming, with heightened glycolysis serving as the central organizer, supported by PPP activation and TCA cycle adaptation. This integrated metabolic network, governed by master regulators including mTOR, AMPK, HIF-1α, c-Myc, PKM2, and T-bet, establishes a permissive microenvironment for autoreactive B cell survival, clonal expansion, and effector differentiation. Critically, this systemic metabolic dysregulation culminates in the production and tissue deposition of nephritogenic autoantibodies, driving the pathogenesis of LN. The presence of metabolically active, pathogenic B cell subsets within the kidney itself—whether in ectopic lymphoid structures or as infiltrating antibody-secreting cells—highlights the direct link between B cell metabolism and intrarenal injury. Preclinical evidence strongly supports the therapeutic targeting of these metabolic pathways. Future translational efforts should prioritize the validation of these mechanisms in well-defined human B cell subsets, the development of selective metabolic modulators with improved pharmacologic profiles, and the advancement of personalized treatment algorithms based on patient-specific metabolic signatures.

## 4 Glucose metabolic reprogramming in renal resident cells

The metabolic reprogramming of infiltrating immune cells, as discussed above, creates a profoundly inflammatory cytokine milieu within the kidney. This microenvironment, in turn, directly impinges upon renal resident cells, driving their own intrinsic metabolic alterations that exacerbate tissue damage. The convergence of immune-driven inflammation and maladaptive metabolic responses in podocytes, mesangial cells, and tubular epithelial cells forms a vicious cycle culminating in the characteristic lesions of LN.

Emerging evidence indicates that glucose metabolic reprogramming in renal resident cells is regulated by specific signaling pathways, contributing to injury in LN and other glomerulopathies. In podocytes, aberrantly glycosylated IgG from LN patients downregulates glycolysis through CaMK4 activation and intracellular calcium dysregulation, reducing PKM2 activity and limiting ATP production. This impairs cytoskeletal integrity and filtration barrier function (137–139). In mesangial cells, LN serum induces DEC2 overexpression, which activates TLR4 signaling and upregulates GLUT1, enhancing glycolytic flux and lactate production (140). DEC2 also promotes proliferation through both glycolysis-dependent PI3K/Akt and glycolysis-independent p38 MAPK/ERK pathways. Combined inhibition of glycolysis 2-DG and MAPK signaling effectively suppresses MC proliferation, highlighting multi-target therapeutic potential (141). In proximal tubular cells, mTORC1 activation drives PPP upregulation via increased glucose-6-phosphate dehydrogenase (G6PD) expression. This leads to NADPH accumulation, NOX2-mediated ROS generation, and tubular apoptosis. mTORC1 inhibition with rapamycin or G6PD knockdown attenuates these effects, suggesting mTORC1-G6PD as a key signaling-metabolic node in tubular injury (11). A recent study based on microdissection of renal biopsy tissues and transcriptomic analysis revealed that in various forms of glomerulonephritis, including LN and ANCA-associated vasculitis, both glomerular and tubulointerstitial compartments exhibit upregulation of the PPP at the transcriptional level. This elevation not only correlates significantly with a decline in glomerular filtration rate but also coincides with increased expression of proinflammatory cytokines such as tumor necrosis factor, suggesting a spatially and functionally close relationship between glucose metabolic reprogramming and localized immune-inflammatory responses (142). Despite these insights, research remains limited. Further studies should explore metabolic cross-talk between cell types, validate human relevance, and evaluate small molecules targeting glycolysis, PPP, or upstream regulators mTORC1, TLR4 and CaMK4 for metabolic reprogramming in kidney diseases.

## 5 Conclusion and prospect

The pathogenesis of SLE and its severe complication, LN, is increasingly being reevaluated through the lens of immunometabolism. As systematically reviewed, glucose metabolic reprogramming is not merely a passive consequence of immune cell activation. Instead, it functions as an active regulatory hub driving functional dysregulation in innate immune cells (monocytes/macrophages, neutrophils, dendritic cells), adaptive immune cells (T cells, B cells), and injury in kidney-resident cells. This paradigm shift not only provides a novel theoretical framework for understanding immune dysregulation in SLE but also opens new therapeutic avenues targeting metabolic pathways.

The translation of metabolic theory into clinical practice is beginning to take shape. Drug repurposing offers the most expedient path. For instance, beyond its immunomodulatory effects, the classic agent hydroxychloroquine has been shown to inhibit glycolysis and the PPP, which may partly explain its long-term benefit in reducing SLE flares (143). Metformin, through AMPK activation and mTOR inhibition, has demonstrated potential in clinical trials to reduce SLE disease activity and steroid dosage, a mechanism linked to correcting the Th17/Treg imbalance and B cell metabolic hyperactivity (144). Rapamycin, which directly targets the core metabolic node mTORC1, has shown encouraging efficacy in patients with refractory SLE and LN, highlighting the critical role of suppressing excessive anabolic metabolism in controlling autoimmunity (123, 145). Currently, these metabolically modulatory drugs are primarily used in clinical practice as adjunctive and steroid-sparing strategies during the active treatment phase. However, whether they can serve as maintenance therapy during remission to regulate the immune system and prevent flares awaits validation from prospective, large-scale studies.

Concurrently, preclinical research is delineating a more precise blueprint for metabolic-targeted therapy. Inhibitors targeting key glycolytic nodes, such as the GLUT1, rate-limiting enzymes HK2/PFKFB3, the multifunctional PKM2 (with both metabolic and transcriptional coactivator functions), LDHA and HIF-1α, can selectively suppress pathogenic Tfh cells, autoreactive B cells, and pro-inflammatory macrophages in lupus animal models without globally compromising immune defense (90, 114, 135). A more innovative approach involves combining metabolic inhibitors with existing biologics (such as anti-CD20, anti-BAFF), aiming to enhance targeted depletion and delay the resurgence of pathogenic cells by transiently disrupting their metabolic adaptability.

Despite the promising outlook, the field faces multiple challenges on its path to maturity. First, many studies reveal associations between metabolic alterations and disease states, whereas definitive proof of a driver role requires more refined genetic and functional experiments. Second, the metabolic state of immune cells may dynamically evolve across different disease stages (active vs. remission) and anatomical compartments, a dynamic landscape that is difficult to capture with the predominantly cross-sectional nature of current research (146, 147). Third, the metabolomic profile in patients is significantly confounded by factors such as concomitant medications (particularly glucocorticoids), comorbidities, and diet, making it essential to disentangle these effects to identify disease-specific metabolic signatures (148). Finally, fundamental pathways like glycolysis are upregulated in nearly all activated lymphocytes. Systemic inhibition may inadvertently impair beneficial subsets such as Tregs that rely on the same pathways, leading to excessive immunosuppression or metabolic toxicity.

First, single-cell multi-omics should be employed to decipher the dynamic metabolic profiles of immune cells in SLE, identifying stage-specific therapeutic targets. Second, non-invasive monitoring tools such as peripheral blood cell-based metabolic activity indices and hyperpolarized ¹³C-pyruvate MRI need to be developed to enable precise patient stratification and treatment response prediction (149, 150). Third, intelligent delivery systems must be designed to enhance the selectivity of metabolic interventions while minimizing impact on normal immune cells. Ultimately, predictive models integrating multidimensional data should be constructed to systematically elucidate metabolic network regulatory mechanisms, providing a theoretical foundation for developing personalized combination therapies.

In summary, the perspective of immunometabolism offers a new map for understanding the complexity of SLE. Targeting glucose metabolic reprogramming represents a therapeutic philosophy aimed at fundamentally modulating immune cell function, holding great promise. However, the success of this path relies on a deep understanding of metabolic heterogeneity, the relentless pursuit of therapeutic selectivity, and the precise implementation of personalized treatment strategies.
